# Supplementary material for: Development of a low-cost culture medium from industrial and environmental by-products for sustainable cultivation of Lactic Acid Bacteria
Source: PLoS One. 2025 Dec 1;20(12):e0337684. doi: 10.1371/journal.pone.0337684 (PMC12668542; doi:10.1371/journal.pone.0337684)
Supplement: S1 Table — (PDF) [file pone.0337684.s001.pdf]

**Parameters**	**Black soldier fly larvae cake**			**Pineapple peel**			**Sugarcane molasses**		
	trial 1	***trial 2***	***trial 3***	***trial 1***	***trial 2***	***trial 3***	***trial 1***	***trial 2***	***trial 3***
**Lipids (% DM)**	16.614	16.280	16.47	0.600	0.750	0.900	0.000	0.000	0.000
**Proteins (% DM)**	48.576	48.570	48.33	4.840	4.890	4.940	7.01	7.020	7.030
**Carbohydrates (% DM)**	26.100	26.400	26.27	8.3600	89.52	89.64	86.79	86.79	86.91
**Na (mg/100g)**	1.112	1.252	1.392	0.613	0.547	0.481	0.504	0.764	1.024
**Mg (mg/100g)**	0.407	0.467	0.437	0.181	0.14	0.138	0.095	0.175	0.255
**K (mg/100g)**	1.235	1.184	1.133	1.466	1.429	1.392	2.822	2.872	2.922
**P (mg/100g)**	0.500	0.710	0.920	0.193	0.136	0.079	0.099	0.067	0.035
**N (mg/100g)**	0.245	0.214	0.183	0.089	0.059	0.029	0.102	0.057	0.012
**Mn (mg/100g)**	0.041	0.017	0.00	0.00	0.017	0.047	0.00	0.006	0.012
**Fe (mg/100g)**	0.067	0.067	0.067	0.145	0.101	0.057	0.088	0.055	0.022
**Zn (mg/100g)**	0.005	0.000	0.053	0.073	0.003	0.000	0.020	0.000	0.004
