# Supplementary material for: Development of a low-cost culture medium from industrial and environmental by-products for sustainable cultivation of Lactic Acid Bacteria
Source: PLoS One. 2025 Dec 1;20(12):e0337684. doi: 10.1371/journal.pone.0337684 (PMC12668542; doi:10.1371/journal.pone.0337684)
Supplement: S2 Table — (PDF) [file pone.0337684.s002.pdf]

| Parameters | Black soldier fly larvae cake (µmol/mL) | Pineapple peel (µmol/mL) | Sugarcane molasses (µmol/mL) |
|---|---|---|---|
| **Histidine** | 5.66 ± 0.02$^{a}$ | 0.026 ± 0.13$^{b}$ | 0.03 ± 0.21$^{b}$ |
| **Serine** | 2.53 ± 0.26$^{a}$ | 0.00 ± 0.00$^{b}$ | 0.03 ± 0.03$^{b}$ |
| **Arginine** | 2.37 ± 0.15$^{a}$ | 0.00 ± 0.00$^{b}$ | 0.00 ± 0.00$^{b}$ |
| **Glycine** | 4.41 ± 0.30$^{a}$ | 0.20 ± 0.01$^{b}$ | 0.15 ± 0.00$^{c}$ |
| **Aspartic acid** | 4.63 ± 0.04$^{a}$ | 0.83 ± 0.21$^{b}$ | 0.76 ± 0.00$^{c}$ |
| **Glutamic acid** | 5.42 ± 0.02$^{a}$ | 0.03 ± 0.12$^{b}$ | 0.02 ± 0.02$^{b}$ |
| **Threonine** | 2.98 ± 0.21$^{a}$ | 0.00 ± 0.00$^{b}$ | 0.00 ± 0.00$^{b}$ |
| **Alanine** | 4.14 ± 0.06$^{a}$ | 0.00 ± 0.00$^{b}$ | 0.04 ± 0.03$^{b}$ |
| **Proline** | 2.27 ± 0.02$^{a}$ | 0.86 ± 0.01$^{b}$ | 0.87 ± 0.01$^{b}$ |
| **Cysteine** | 2.09 ± 0.41$^{a}$ | 0.00 ± 0.00$^{b}$ | 0.00 ± 0.012$^{b}$ |
| **Lysine** | 0.89 ± 0.31$^{c}$ | 1.99 ± 0.17$^{a}$ | 1.73 ± 0.01$^{b}$ |
| **Tyrosine** | 2.78 ± 0.14$^{a}$ | 0.00 ± 0.00$^{b}$ | 0.00 ± 0.00$^{b}$ |
| **Methionine** | 0.21 ± 0.20$^{a}$ | 0.00 ± 0.00$^{b}$ | 0.00 ± 0.00$^{b}$ |
| **Valine** | 2.79 ± 0.01$^{a}$ | 0.00 ± 0.00$^{b}$ | 0.00 ± 0.00$^{b}$ |
| **Isoleucine** | 1.70 ± 0.03$^{a}$ | 0.00 ± 0.00$^{b}$ | 0.00 ± 0.00$^{b}$ |
| **Leucine** | 3.21 ± 0.01$^{a}$ | 0.00 ± 0.00$^{b}$ | 0.00 ± 0.00$^{b}$ |
| **Phenylalanine** | 1.90 ± 0.20$^{a}$ | 0.00 ± 0.00$^{b}$ | 0.00 ± 0.00$^{b}$ |

$^{a-c}$: Values with different letters on the same line differ significantly ($p < 0.05$).
