# Supplementary material for: Development of a low-cost culture medium from industrial and environmental by-products for sustainable cultivation of Lactic Acid Bacteria
Source: PLoS One. 2025 Dec 1;20(12):e0337684. doi: 10.1371/journal.pone.0337684 (PMC12668542; doi:10.1371/journal.pone.0337684)
Supplement: S3 Table — (PDF) [file pone.0337684.s003.pdf]

Parameters	Black soldier fly larvae cake (µmol/mL)			Pineapple peel (µmol/mL)			Sugarcane molasses (µmol/mL)		
	trial 1	*trial 2*	*trial 3*	*trial 1*	*trial 2*	*trial 3*	*trial 1*	*trial 2*	*trial 3*
**Histidine**	5.64	5.66	5.68	0.00	0.026	0.056	0.00	0.03	0.24
**Serine**	2.27	2.53	2.79	0.00	0.00	0.00	0.00	0.03	0.06
**Arginine**	2.22	2.37	2.52	0.00	0.00	0.00	0.00	0.00	0.00
**Glycine**	4.11	4.41	4.71	0.19	0.20	0.21	0.15	0.15	0.15
**Aspartic acid**	4.59	4.63	4.67	0.62	0.83	1.04	0.76	0.76	0.76
**Glutamic acid**	5.40	5.42	5.44	0.00	0.03	0.06	0.00	0.02	0.04
**Threonine**	2.77	2.98	3.19	0.00	0.00	0.00	0.00	0.00	0.00
**Alanine**	4.08	4.14	4.20	0.00	0.00	0.00	0.01	0.04	0.07
**Proline**	2.25	2.27	2.29	0.85	0.86	0.87	0.86	0.87	0.88
**Cysteine**	1.68	2.09	2.50	0.00	0.00	0.00	0.00	0.00	0.00
**Lysine**	0.58	0.89	1.20	1.82	1.99	2.16	1.72	1.73	1.74
**Tyrosine**	2.64	2.78	2.92	0.00	0.00	0.00	0.00	0.00	0.00
**Methionine**	0.01	0.21	0.41	0.00	0.00	0.00	0.00	0.00	0.00
**Valine**	2.78	2.79	2.80	0.00	0.00	0.00	0.00	0.00	0.00
**Isoleucine**	1.67	1.70	1.73	0.00	0.00	0.00	0.00	0.00	0.00
**Leucine**	3.20	3.21	3.22	0.00	0.00	0.00	0.00	0.00	0.00
