# Supplementary material for: Development of a low-cost culture medium from industrial and environmental by-products for sustainable cultivation of Lactic Acid Bacteria
Source: PLoS One. 2025 Dec 1;20(12):e0337684. doi: 10.1371/journal.pone.0337684 (PMC12668542; doi:10.1371/journal.pone.0337684)
Supplement: S5 Table — (PDF) [file pone.0337684.s005.pdf]

**Trials**	**BCH (%v/v)**	**PPH (%v/v)**	**SM (%v/v)**	***Lactiplantibacillus plantarum* 5602** (log CFU/mL)			***Lacticaseibacillus rhamnosus* 347** (log CFU/mL)			***Lactobacillus acidophilus* 291** (log CFU/mL)			***Lactobacillus gasseri* 5359** (log CFU/mL)			***Bifidobacterium bifidum 231*** (log CFU/mL)		
				trial 1	***trial 2***	***trial 3***	***trial 1***	***trial 2***	***trial 3***	***trial 1***	***trial 2***	***trial 3***	***trial 1***	***trial 2***	***trial 3***	***trial 1***	***trial 2***	***trial 3***
**1**	62.500	15.625	21.875	9.28	9.35	9.42	8.93	9.63	10.33	9.46	9.66	9.86	9.39	9.60	9.81	9.32	9.64	9.96
**2**	56.250	21.875	21.875	9.21	9.33	9.45	9.05	9.65	10.25	9.60	9.72	9.84	9.50	9.67	9.84	9.51	9.63	9.75
**3**	68.750	15.625	15.625	9.17	9.19	9.21	9.03	9.43	9.83	9.53	9.71	9.89	9.22	9.60	9.98	9.09	9.53	9.97
**4**	50.000	25.000	25.000	9.21	9.31	9.41	9.29	9.62	9.95	9.57	9.67	9.77	10.06	9.65	9.24	9.51	9.63	9.57
**5**	62.500	18.750	18.750	9.26	9.36	9.46	9.41	9.69	9.97	9.33	9.73	10.13	9.45	9.68	9.91	9.36	9.64	9.92
**6**	62.500	12.500	25.000	9.22	9.27	9.32	9.26	9.51	9.76	9.25	9.60	9.95	9.17	9.58	9.99	9.48	9.65	9.82
**7**	62.500	25.000	12.500	9.14	9.24	9.34	9.07	9.47	9.87	9.74	9.57	9.40	9.38	9.63	9.88	9.37	9.58	9.79
**8**	62.500	21.875	15.625	9.26	9.33	9.40	9.49	9.62	9.75	9.93	9.65	9.37	9.50	9.61	9.72	9.60	10.00	9.20
**9**	75.000	12.500	12.500	9.01	9.04	9.07	9.11	9.38	9.63	9.22	9.82	9.52	9.22	9.54	9.86	9.51	9.52	9.53
**MRS/ M17**				8.96	9.2	9.44	9.54	9.69	9.84	9.62	9.75	9.49	9.39	9.59	9.79	9.82	9.53	9.24

S7 Table (next)

**Trials**	**BCH (%v/v)**	**PPH (%v/v)**	**SM (%v/v)**	***Bacillus subtilis* 215** (log CFU/mL)			***Lactobacillus delbrueckii* subsp. *bulgaricus* 293** (log CFU/mL)			***Streptococcus thermophilus* 295** (log CFU/mL)			***Lactococcus lactis* subsp. *lactis* MA2** (log CFU/mL)			***Lactococcus lactis* subsp. *lactis* MF5** (log CFU/mL)		
				trial 1	***trial 2***	***trial 3***	***trial 1***	***trial 2***	***trial 3***	***trial 1***	***trial 2***	***trial 3***	***trial 1***	***trial 2***	***trial 3***	***trial 1***	***trial 2***	***trial 3***
**1**	62.500	15.625	21.875	9.48	9.58	9.68	9.53	9.60	9.67	9.34	9.64	9.94	9.28	9.41	9.54	9.45	9.55	9.65
**2**	56.250	21.875	21.875	9.57	9.60	9.63	9.53	9.67	9.71	9.45	9.63	9.61	9.21	9.43	9.55	9.49	9.59	9.69
**3**	68.750	15.625	15.625	9.42	9.56	9.70	9.57	9.60	9.63	9.39	9.50	9.72	9.31	9.41	9.51	9.34	9.44	9.54
**4**	50.000	25.000	25.000	9.40	9.57	9.74	9.45	9.63	9.81	9.34	9.31	9.53	9.25	9.42	9.59	9.43	9.52	9.61
**5**	62.500	18.750	18.750	9.42	9.63	9.84	9.57	9.65	9.73	9.41	9.64	9.72	9.35	9.48	9.61	9.45	9.55	9.65
**6**	62.500	12.500	25.000	9.50	9.57	9.64	9.53	9.63	9.69	9.28	9.33	9.60	9.36	9.42	9.48	9.39	9.55	9.71
**7**	62.500	25.000	12.500	9.46	9.56	9.66	9.33	9.53	9.73	9.50	9.54	9.58	9.21	9.31	9.41	9.38	9.51	9.64
**8**	62.500	21.875	15.625	9.57	9.57	9.57	9.56	9.59	9.62	9.38	9.58	9.58	9.30	9.34	9.38	9.50	9.54	9.58
**9**	75.000	12.500	12.500	9.48	9.52	9.56	9.41	9.52	9.63	9.40	9.41	9.41	8.83	8.92	9.01	9.32	9.40	9.48
**MRS/ M17**				9.54	9.54	9.54	9.30	9.55	9.70	9.23	9.31	9.39	8.94	9.12	9.32	9.37	9.49	9.61
