# Supplementary material for: Development of a low-cost culture medium from industrial and environmental by-products for sustainable cultivation of Lactic Acid Bacteria
Source: PLoS One. 2025 Dec 1;20(12):e0337684. doi: 10.1371/journal.pone.0337684 (PMC12668542; doi:10.1371/journal.pone.0337684)
Supplement: S6 Table — (PDF) [file pone.0337684.s006.pdf]

Responses (Biomass)	R^2 (%)	AMDA	Bf
***Lactiplantibacillus plantarum* 5602**	89.27	0.02	1.06
***Lacticaseibacillus rhamnosus* 347**	91.86	0.00	1.03
***Lactobacillus acidophilus* 291**	90.28	0.04	1
***Lactobacillus gasseri* 5359**	85.95	0.03	1.01
Lactobacillus delbrueckii* subsp. *bulgaricus	90.62	0.02	1.01
***Streptococcus thermophilus* 295**	95.76	0.01	1.00
***Lactococcus lactis* subsp. *lactis* MA2**	93.05	0.00	1.02
***Lactococcus lactis* subsp. *lactis* MF5**	91.05	0.01	1
***Bifidobacterium bifidum* 231**	90.82	0.02	1.07
***Bacillus subtilis* 215**	83.18	0.03	1.00

R^2: Coefficient of determination. **AADM:** Absolute Mean Deviation Analysis. **Bf:** Bias factor
