# Supplementary material for: Development of a low-cost culture medium from industrial and environmental by-products for sustainable cultivation of Lactic Acid Bacteria
Source: PLoS One. 2025 Dec 1;20(12):e0337684. doi: 10.1371/journal.pone.0337684 (PMC12668542; doi:10.1371/journal.pone.0337684)
Supplement: S7 Table — (PDF) [file pone.0337684.s007.pdf]

**Strains**	**Optimal experimental biomass values** (log CFU/mL)			**Optimal experimental biomass values** (log CFU/mL)		
	trial 1	***trial 2***	***trial 3***	***trial 1***	***trial 2***	***trial 3***
***Lactiplantibacillus plantarum* 5602**	8.88	9.43	9.98	9.29	9.39	9.49
***Lacticaseibacillus rhamnosus* 347**	9.53	9.77	10.1	9.62	9.70	9.78
***Lactobacillus acidophilus* 291**	9.85	9.86	9.87	9.52	9.74	9.96
***Lactobacillus gasseri* 5359**	9.49	9.58	9.67	9.52	9.67	9.82
***Lactobacillus delbrueckii* subsp. *bulgaricus* 293**	9.70	9.70	9.70	9.59	9.68	9.77
***Streptococcus thermophilus* 295**	9.57	9.67	9.77	9.51	9.62	9.73
***Lactococcus lactis* subsp. *lactis* MA2**	9.11	9.47	9.83	9.46	9.51	9.73
***Lactococcus lactis* subsp. *lactis* MF5**	9.12	9.52	9.92	9.47	9.57	9.67
***Bifidobacterium bifidum* 231**	9.11	9.65	10.19	9.48	9.61	9.74
***Bacillus subtilis* 215**	9.54	9.60	9.66	9.43	9.62	9.81
