# Supplementary material for: Development of a low-cost culture medium from industrial and environmental by-products for sustainable cultivation of Lactic Acid Bacteria
Source: PLoS One. 2025 Dec 1;20(12):e0337684. doi: 10.1371/journal.pone.0337684 (PMC12668542; doi:10.1371/journal.pone.0337684)
Supplement: S8 Table — (PDF) [file pone.0337684.s008.pdf]

Trials	BCH (% v/v)	PPH (% v/v)	SM (% v/v)	***Lactiplantibacillus plantarum*** **5602** / *S. aureus NCDC* 109			***Lacticaseibacillus rhamnosus*** **347 /** *S. aureus NCDC* 109			**Lactococcus lactis subsp. *lactis* MA2 /** *Pediococcus acidilactici*		
				**Inhibition zone diameter (mm)**								
				trial 1	***trial 2***	***trial 3***	***trial 1***	***trial 2***	***trial 3***	***trial 1***	***trial 2***	***trial 3***
**1**	62.500	15.625	21.8750	10.22	10.50	10.78	9.58	10.0	9.58	13.56	13.70	13.84
**2**	56.250	21.875	21.875	10.50	10.50	10.50	9.59	10.0	9.59	12.9	13.00	13.10
**3**	68.750	15.625	15.625	9.80	10.00	10.20	9.50	9.50	9.50	12.8	12.50	12.20
**4**	50.000	25.000	25.000	10.32	10.50	10.68	9.21	9.50	9.21	10.8	13.00	13.20
**5**	62.500	18.750	18.750	11.10	11.50	11.90	11.13	11.50	11.13	13.10	14.00	14.90
**6**	62.500	12.500	25.000	9.80	10.50	11.20	10.70	10.70	10.70	13.43	13.50	13.57
**7**	62.500	25.000	12.500	8.59	9.00	9.41	8.58	9.00	8.58	11.8	11.50	11.70
**8**	62.500	21.875	15.625	8.80	9.50	10.20	8.80	9.50	8.80	11.9	12.00	12.10
**9**	75.000	12.500	12.500	7.00	7.00	7.00	6.93	7.00	6.93	8.93	9.00	9.10
**MRS/M17**				10.89	11.11	11.00	11.23	11.5	11.23	13.60	14.00	14.40

S8 Table (next)

**Trials**	BCH (% v/v)	PPH (% v/v)	SM (% v/v)	***Lactococcus lactis* subsp. *lactis* MF5** / *Pediococcus acidilactici*			***Bifidobacterium bifidum*** / *E. coli* ATCC 11775		
				**Inhibition zone diameter (mm)**					
				trial 1	***trial 2***	***trial 3***	***trial 1***	***trial 2***	***trial 3***
**1**	62.500	15.625	21.8750	13.35	13.50	13.65	10.85	11.00	11.15
**2**	56.250	21.875	21.875	13.93	14.00	14.07	10.89	11.00	11.11
**3**	68.750	15.625	15.625	11.80	11.50	11.70	9.10	10.50	11.40
**4**	50.000	25.000	25.000	12.80	13.00	13.20	10.22	10.50	10.78
**5**	62.500	18.750	18.750	12.93	13.00	13.07	11.33	11.50	11.67
**6**	62.500	12.500	25.000	13.33	13.40	13.47	9.80	10.50	11.25
**7**	62.500	25.000	12.500	11.80	12.00	12.20	8.30	9.00	9.70
**8**	62.500	21.875	15.625	12.34	12.50	12.66	9.50	9.50	9.50
**9**	75.000	12.500	12.500	9.93	10.00	10.07	7.58	8.00	8.42
**MRS/M17**				13.87	14.00	14.13	10.63	11.0	11.37
