# Supplementary material for: Development of a low-cost culture medium from industrial and environmental by-products for sustainable cultivation of Lactic Acid Bacteria
Source: PLoS One. 2025 Dec 1;20(12):e0337684. doi: 10.1371/journal.pone.0337684 (PMC12668542; doi:10.1371/journal.pone.0337684)
Supplement: S9 Table — (PDF) [file pone.0337684.s009.pdf]

**Responses (Bacteriocin production)**	**R^2 (%)**	**AMDA**	**Bf**
***Lactiplantibacillus plantarum* 5602**	87.43	0.00	1.03
***Lacticaseibacillus rhamnosus* 347**	85.82	0.0	0.9
***Lactococcus lactis* subsp. *lactis* MA2**	90.91	0.01	1.00
***Lactococcus lactis* subsp. *lactis* MF5**	96.26	0.02	1.05
***Bifidobacterium bifidum* 231**	88.01	0.01	1.04

R^2: Coefficient of determination. **AMDA:** Absolute Mean Deviation Analysis. **Bf:** Bias factor.
