# Supplementary material for: Development of a low-cost culture medium from industrial and environmental by-products for sustainable cultivation of Lactic Acid Bacteria
Source: PLoS One. 2025 Dec 1;20(12):e0337684. doi: 10.1371/journal.pone.0337684 (PMC12668542; doi:10.1371/journal.pone.0337684)
Supplement: S10 Table — (PDF) [file pone.0337684.s010.pdf]

**Strains**	**Experimental optimal activity values (mm)**			**Predicted optimal activity values (mm)**		
	trial 1	***trial 2***	***trial 3***	***trial 2***	***trial 2***	***trial 3***
***Lactiplantibacillus plantarum* 5602**	11.91	11.70	11.49	11.73	11.83	11.93
***Lacticaseibacillus rhamnosus* 347**	11.60	11.70	11.80	11.45	11.35	11.40
***Lactococcus lactis* subsp. *lactis* MA2**	14.71	14.50	14.29	14.59	14.81	14.70
***Lactococcus lactis* subsp. *lactis* MF5**	13.99	14.10	14.21	14.24	14.32	14.40
***Bifidobacterium bifidum* 231**	11.77	11.99	12.03	11.98	12.02	12.00
