# Supplementary material for: Development of a low-cost culture medium from industrial and environmental by-products for sustainable cultivation of Lactic Acid Bacteria
Source: PLoS One. 2025 Dec 1;20(12):e0337684. doi: 10.1371/journal.pone.0337684 (PMC12668542; doi:10.1371/journal.pone.0337684)
Supplement: S11 Table — (PDF) [file pone.0337684.s011.pdf]

Parameters	Soldier fly larvae cake hydrolysate			Pineapple peel hydrolysate			Sugarcane molasses		
	trial 1	*trial 2*	*trial 3*	*trial 1*	*trial 2*	*trial 3*	*trial 1*	*trial 2*	*trial 3*
**Proteins (%DM)**	3.13	3.19	3.16	1.98	1.94	2.02	0.10	0.28	0.46
**Lipids (%DM)**	0.08	0.03	0.13	0.07	0.15	0.23	0.00	0.00	0.00
**Carbohydrates (% DM)**	3.55	3.47	3.39	5.28	5.31	5.34	3.42	3.47	3.52
**Ash (%DM)**	1.00	1.26	1.13	0.37	0.31	0.34	0.19	0.24	0.29
