# Supplementary material for: Development of a low-cost culture medium from industrial and environmental by-products for sustainable cultivation of Lactic Acid Bacteria
Source: PLoS One. 2025 Dec 1;20(12):e0337684. doi: 10.1371/journal.pone.0337684 (PMC12668542; doi:10.1371/journal.pone.0337684)
Supplement: Attachment — Submitted filename: Comments.pdf [file pone.0337684.s013.pdf]

The following comments are to improve the quality of your research paper:

Introduction section:

Describe more the characteristics or importance of the 3 different sources of the culture medium. In abbreviations, the name or meaning is placed first, and then the corresponding abbreviation in parentheses, and you are interchanged in line 44

Materials and methods section:

Check the names of the strains because they are misspelled, for example, Lactoccocus is *Lactococcus*, also the term Mc Farland is McFarland, an example is also in line 97, and in the tables, check each one well.

Improve in general, all the titles of the tables so that they are more descriptive and punctual, because some are too long, and also check the titles of the columns, because some have a capital initial and others do not

In Table 1 the term status is the function as described in the title, and the case of technological is the application of the strain

Try to simplify the methodologies for example autoclaved (121°C, 15psi), in the sequence of the pineapple peel hydrolysate, the wording is confusing, there is a lack of volume of water that is used, there is a lack of filters, check also in the black soldier fly larvae cake the equipment is missing team names, volumes and in the solutions the name of the reagent is placed first and then the concentration

Check the number of subtitles. The black soldier fly larvae cake is reserved for later use. It is not specified how they keep it, and they do not mention anything from the number 3 source. Try to be more orderly in writing, follow a sequence in terms of the components of the medium throughout the article try to handle that sequence to describe them

In line 112, reference is made to the IUPAC and AOAC methods for determining the nutritional profile of the sample, but it is poorly worded to mention what parameters were measured and with what technique, respectively, because it remains as an idea in the air. Because next comes the quantification of minerals, but it is by another type of analysis, according to the wording

Line 125 goes first with the name of the acid, followed by the concentration

Table 2: Homogenize the initial in all column titles

Improve the wording of the title of table 3, it is very long, you can use a table note or better describe it in the text

Line 180 error in reagent name

In design, it is a mixture design or factorial design

Results section

It is recommended to divide the information into more concise sentences, grouping results by category, for example, maximum values compared to the control. In the text, use different words for significantly, use variants such as notably superior, markedly increased, etc., or place it in the tables of results or methods section.

It is suggested to create a table with the strain, range (CFU/ml), max vs control, p-value, and write a paragraph that highlights only the key findings to make comparisons clearer in the text

Check again in this section the names of the strains and units

Explain a little more what the coefficients mean; they only mention linear effects and interactions. This can help in the interpretation to see if there is inhibition or stimulation

The note in Table 9 is incomplete

Discussion Section:

The section presents a logical structure and supported argumentation, however there are disconnections with the results and superficial interpretations, for example the result is described but in the discussion the acceptable rank is not specified if it is or not, if it is better or not without comparative values, in the terms more similar vs equal or higher there is contradiction when reading them, In the interpretation of mathematical models as mentioned before there is no interpretation of the coefficients to carry out the discussion in this section. The section is totally positive, but what happens with the non-optimal results, because it can be this result, the variability between replicates, the stability of the environment at room temperature, or useful life, with its results, it could be scaled, the cost-benefit
