# Supplementary material for: Development of a low-cost culture medium from industrial and environmental by-products for sustainable cultivation of Lactic Acid Bacteria
Source: PLoS One. 2025 Dec 1;20(12):e0337684. doi: 10.1371/journal.pone.0337684 (PMC12668542; doi:10.1371/journal.pone.0337684)
Supplement: Attachment — Submitted filename: Response to Reviewers.docx [file pone.0337684.s014.docx]

Rebuttal Letter

Article "**Development of a low-cost culture medium from industrial and environmental by-products for sustainable cultivation of Lactic Acid Bacteria**"

Dear Academic Editor and Reviewers,

We would like to sincerely thank the Editor and both Reviewers for their valuable time, constructive comments, and insightful suggestions. These remarks have greatly helped us to improve the clarity, depth, and scientific quality of our manuscript. Below, we provide a detailed, point-by-point response to each comment, preceded by an R:.

Journal Requirements

When submitting your revision, we need you to address these additional requirements.

**1. Please ensure that your manuscript meets PLOS ONE's style requirements, including those for file naming. The PLOS ONE style templates can be found at** [**https://journals.plos.org/plosone/s/file?id=wjVg/PLOSOne_formatting_sample_main_body.pdf**](https://journals.plos.org/plosone/s/file?id=wjVg/PLOSOne_formatting_sample_main_body.pdf)**and**[**https://journals.plos.org/plosone/s/file?id=ba62/PLOSOne_formatting_sample_title_authors_affiliations.pdf**](https://journals.plos.org/plosone/s/file?id=ba62/PLOSOne_formatting_sample_title_authors_affiliations.pdf)

R: The manuscript has been adapted to meet the PLOS ONE’s formatting standards, following the provided templates for structure, tables, and figure captions.

**2. We note that your Data Availability Statement is currently as follows: All relevant data are within the manuscript and its Supporting Information files.**

**Please confirm at this time whether or not your submission contains all raw data required to replicate the results of your study. Authors must share the “minimal data set” for their submission. PLOS defines the minimal data set to consist of the data required to replicate all study findings reported in the article, as well as related metadata and methods (https://journals.plos.org/plosone/s/data-availability#loc-minimal-data-set-definition).**

**For example, authors should submit the following data:**

**- The values behind the means, standard deviations and other measures reported;
- The values used to build graphs;**

**- The points extracted from images for analysis.**

**Authors do not need to submit their entire data set if only a portion of the data was used in the reported study.**

**If your submission does not contain these data, please either upload them as Supporting Information files or deposit them to a stable, public repository and provide us with the relevant URLs, DOIs, or accession numbers. For a list of recommended repositories, please see https://journals.plos.org/plosone/s/recommended-repositories.**

**If there are ethical or legal restrictions on sharing a de-identified data set, please explain them in detail (e.g., data contain potentially sensitive information, data are owned by a third-party organization, etc.) and who has imposed them (e.g., an ethics committee). Please also provide contact information for a data access committee, ethics committee, or other institutional body to which data requests may be sent. If data are owned by a third party, please indicate how others may request data access.**

R: Thanks for your observation. To comply with the Data Availability Statement, all the raw data required to reproduce the results (including means, and standard deviations) have been uploaded as Supporting Information files (S4–S11 Tables).

**3. We note you have included a table to which you do not refer in the text of your manuscript. Please ensure that you refer to Table 9 in your text; if accepted, production will need this reference to link the reader to the Table.**

R: We thank the reviewer for this observation. This concern was addressed in the revised manuscript while the initial Table 9 has become Table 8 and referred in the text.

See Lines 433 – 436_Revised manuscript unmarked: “compared to the predicted ones, as shown in Table 8. Overall, the experimental optimal biomass values obtained do not differ significantly (p˂0.05) from the predicted ones, with a desirability of 0.95, thus validating the biomass values predicted by the software.

Table 8. By-product proportions of the compromise medium and validation of biomass production model”

**4. If the reviewer comments include a recommendation to cite specific previously published works, please review and evaluate these publications to determine whether they are relevant and should be cited. There is no requirement to cite these works unless the editor has indicated otherwise. [Note: HTML markup is below. Please do not edit.]**

R: None of the reviewers recommended to cite specific previously published works.

Reviewer #1

**1. The introduction is well-written but repeats general LAB information that is already well-known. This can be shortened, and more space can be dedicated to gaps in existing low-cost media studies.**

R: The introduction has been revised to reduce general background on lactic acid bacteria and to highlight research gaps and recent advances in low-cost media development. New references have been added (Acosta-Piantini *et al.* (2023) and Valle Vargas *et al*., 2024) to emphasize on the sustainability-oriented strategies in medium formulation.

See Line 65 – 69_Revised manuscript unmarked: “Similarly, Valle Vargas et al. [13] demonstrated that media formulated from whey, sugarcane molasses, and palm kernel cake could support the growth of fish probiotics *Lactococcus lactis* A12, *Priestia megaterium* M4, and *Priestia* sp. M10, while Acosta-Piantini et al. [14] showed that hydrolysed sugarcane molasses could serve as an economical substrate for mass production of *Lacticaseibacillus paracasei*”

**2. Minor grammatical errors occur, such as article misuse (“a medium” vs. “the medium”).**

R: All grammatical inconsistencies and article misuses have been corrected throughout the manuscript to improve precision and readability.

**3. The phrase “black soldier fly larvae cake” is repeated very frequently consider abbreviation (BSFLC) after first mention.**

R: We thank the reviewer for this constructive suggestion. The term “black soldier fly larvae cake” has been abbreviated as “BC” after its first mention throughout the text, tables, and figure captions. The choice of BC instead of BSFLC was to comply with the already existing abbreviations such as BCH in the text, tables, figures and their captions and footnotes.

**4. Why does the author only consider growth measurements by plate counts (CFU/mL)? Inclusion of growth curves (OD600 vs. time), pH reduction, or lactic acid concentration would improve medium assessment.**

R: Thank to the reviewer for this insightful observation. The purpose of growth measurements by plate count was to have an idea of viable biomass, as the strains are targeted to be used for their industrial or probiotic applications and must be alive. Authors like Valle Vargas *et al.* (2024) cited in our work have also considered measuring viable count at the initial and end of incubation time as a good way to express biomass production in the formulated and control media, Nevertheless, we agree with the reviewer that evaluating other kinetic parameters over time could provide specific information on lag phase, log phase, generation time, etc… But, given the density of results at this phase, we taught to evaluate these kinetic parameters in the next phase that will focus on optimising the strains growth conditions in the compromise medium.

**5. The amino acid profile of the by-products (Table 5) is supportive but not central. It could be summarized.**

R: We thank the reviewer for this suggestion that will reduce the length of the manuscript. The full amino acid profile has been moved to Supplementary Information (S1 Table), and a short summary remains in the Results section:

See Line 255_Revised manuscript unmarked: “The amino acid profile of the three by-products (S1 Table) shows that……..”

**6. Table 8 – Validation of Mathematical Models for Biomass Production: These details could be summarized.**

R: As suggested by the reviewer, the detailed model validation parameters (R², AMDA, Bf) for biomass and bacteriocin productions have been transferred to Supporting information (S2 and S3 Tables. The Results section now includes a concise statement. See Lines 367-368 and 505-507_Revised manuscript unmarked:

Reviewer #2

**1. Describe more the characteristics or importance of the 3 different sources of the culture medium. In abbreviations, the name or meaning is placed first, and then the corresponding abbreviation in parentheses, and you are interchanged in line 44**

R: Thanks for this constructive suggestion. Statements with interchanged abbreviations were canceled due to another reviewer suggestion. Elsewhere, we expanded the introduction to include a clearer description of each by-product.

See Lines 75-85_Revised manuscript unmarked: “The black soldier fly larvae *(Hermetia illucens)* cake, a protein-rich residue from the biofuel and animal feed industries, represents an emerging biotechnological resource in Africa. It contains high-quality proteins, lipids, minerals (Fe, Zn, Mg), and bioactive compounds which can stimulate microbial growth and metabolism [15]. Pineapple peels, abundantly generated by fruit juice industries and local markets, are rich in fermentable sugars (glucose, fructose, sucrose), organic acids, and B-group vitamins, as well as phenolic compounds that may enhance bacterial stress resistance [16]. Sugarcane molasses, a by-product of sugar refineries such as SOSUCAM in Cameroon, provides readily assimilable carbohydrates, minerals (Ca, K, Mg), and trace elements essential for enzymatic functions in LAB [17]. Their combination may allow the development of economical and sustainable culture media in line with the principles of the circular bioeconomy”

**2. (Materials and Methods section): Check the names of the strains because they are misspelled, for example, Lactoccocus is *Lactococcus*, also the term Mc Farland is McFarland, an example is also in line 97, and in the tables, check each one well.**

R: Thanks for this recall. All strain names and technical terms have been double checked and corrected, when necessary, throughout the text and tables.

**3. (Materials and Methods section): Improve in general, all the titles of the tables so that they are more descriptive and punctual, because some are too long, and also check the titles of the columns, because some have a capital initial and others do not**

R: We thank the reviewer for this helpful suggestion. All table titles have been revised for conciseness and clarity throughout the manuscript. Column headings were standardized, and consistent capitalization was applied throughout.

See Lines 95; 167; 173; 253; 266; 304; 320; 436; 459; 474; 547; 555_Revised manuscript unmarked:

**4. (Materials and Methods section): In Table 1 the term status is the function as described in the title, and the case of technological is the application of the strain**

R: In Table 1, the term applications has been added to the column heading, as well as in the title.

See Lines 95_Revised manuscript unmarked:

**5. (Materials and Methods section):Try to simplify the methodologies for example autoclaved (121°C, 15psi), in the sequence of the pineapple peel hydrolysate, the wording is confusing, there is a lack of volume of water that is used, there is a lack of filters, check also in the black soldier fly larvae cake the equipment is missing team names, volumes and in the solutions the name of the reagent is placed first and then the concentration**

Thanks for this valuable suggestion. These concerns were properly addressed. Additionnal equipment, sterilisation pressure and water volume were added. Some sentences were also rephrased.

See Lines 118-176_Revised manuscript unmarked:

**6. (Materials and Methods section): Check the number of subtitles. The black soldier fly larvae cake is reserved for later use. It is not specified how they keep it, and they do not mention anything from the number 3 source. Try to be more orderly in writing, follow a sequence in terms of the components of the medium throughout the article try to handle that sequence to describe them**

R: We thank the reviewer for this helpful suggestion. The subtitles/subheadings have been fixed to maximum 3 levels. We also improve the readability and flow of this section as well as we maintained the same sequence of the medium component’s terms throughout the manuscript.

See Materials and Methods, Results and Discussion sections_Revised manuscript unmarked:

**7. (Materials and Methods section): In line 112, reference is made to the IUPAC and AOAC methods for determining the nutritional profile of the sample, but it is poorly worded to mention what parameters were measured and with what technique, respectively, because it remains as an idea in the air. Because next comes the quantification of minerals, but it is by another type of analysis, according to the wording**

R: Thanks for bringing this important point to our attention. This statement has been rewritten while providing which parameter was measured with what techniques

See Lines 118-120_Revised manuscript unmarked: “The physico-chemical analysis of pineapple peels, BC and sugarcane molasses was conducted according to IUPAC methods [20] for fats and sugars, AOAC methods [21] for proteins and ash.”

**7. (Materials and Methods section): Line 125 goes first with the name of the acid, followed by the concentration**

R: This has been corrected accordingly.

See Line 133_Revised manuscript unmarked: “Samples were first hydrolysed using hydrochloric acid (HCl, 6N)….”

**8. (Materials and Methods section): Table 2: Homogenize the initial in all column titles**

R: This has been corrected in Table 2 and all the other Tables of the Manuscript.

See Lines 95; 167; 173; 253; 266; 304; 320; 436; 459; 474; 547; 555_Revised manuscript unmarked:

**9. (Materials and Methods section): Improve the wording of the title of table 3, it is very long, you can use a table note or better describe it in the text**

R: The wording of Table 3 has been improved, as well as that of the other Tables as per the suggestions of the other Reviewer. See Line 173_Revised manuscript unmarked:

**10. (Materials and Methods section): Line 180 error in reagent name**

R: This has been corrected as NaOH (6N). See Line 158_Revised manuscript unmarked:

**11. (Materials and Methods section): In design, it is a mixture design or factorial design**

R: It is a mixture design as stated at section Materials and method.

**12. (Results section): It is recommended to divide the information into more concise sentences, grouping results by category, for example, maximum values compared to the control. In the text, use different words for significantly, use variants such as notably superior, markedly increased, etc., or place it in the tables of results or methods section.**

R: We thank the reviewer for this valuable suggestion. The Results section has been reorganized for better readability and flow. See Lines 242-566_Revised manuscript unmarked:

**13. (Results section): It is suggested to create a table with the strain, range (CFU/ml), max vs control, p-value, and write a paragraph that highlights only the key findings to make comparisons clearer in the text**

R: We thank the reviewer for this constructive suggestion. New summary tables (Tables 7 and 10) were added, presenting CFU/mL ranges, maximum growth values, and statistical comparisons (p-values) between experimental and the control (MRS/M17) media.

See Lines 320 and 474_Revised manuscript unmarked:

**14. (Results section): Check again in this section the names of the strains and units**

R: We thank the reviewer for these valuable observations**.** All strain names have been carefully double checked and corrected, when necessary, throughout the manuscript according to the official taxonomic nomenclature. Measurement units (log CFU/mL, mm, %, etc….) have also been standardised across tables and text to ensure consistency and clarity.

**15. (Results section): Explain a little more what the coefficients mean; they only mention linear effects and interactions. This can help in the interpretation to see if there is inhibition or stimulation**

R: Thanks for this observation. We have expanded the interpretation in the Discussion as:

“For all the strains, the SM (x_3_), the interaction of BCH and PPH (x_1_x_2_), as well as the interaction of PPH and SM (x_2_x_3_) showed positive effects on biomass production*,* except for *Lacticaseibacillus rhamnosus* 347. In addition, the linear effect of BCH (x_1_) was also positive on biomass production of *Lactobacillus gasseri* 5359, *Streptococcus thermophilus* 295, *Bifidobacterium bifidum* 231, *Bacillus subtilis* 215, *Lactobacillus delbrueckii* subsp. *bulgaricus 293*, and *Lactococcus lactis* subsp. *lactis* MA2 and MF5. This indicates that BCH and SM stimulated biomass production of the strains, while PPH did not contributed to this enhanced production. In addition to these positive linear effects, the positive interactions (x_1_x_2_ and x_2_x_3_,) observed for these by-products suggests that they act in synergy to enhance bacterial growth.”

See Lines 352-361_Revised manuscript unmarked:

**16. (Results section): The note in Table 9 is incomplete**

R: Thanks for this remark. The footnote in Table 9, which is now Table 8 in the revised manuscript, has been completed for clarity.

See Lines 436-437_Revised manuscript unmarked: “^a-a^ In the same line, means with different lower-case letters differ significantly (p < 0.05). BCH: black soldier fly larvae cake hydrolysate (% v/v); PPH: pineapple peel hydrolysate (% v/v); SM: 4% sugarcane molasses (% v/v)”.

**17. (Discussion section): The section presents a logical structure and supported argumentation, however there are disconnections with the results and superficial interpretations, for example the result is described but in the discussion the acceptable rank is not specified if it is or not, if it is better or not without comparative values, in the terms more similar vs equal or higher there is contradiction when reading them, In the interpretation of mathematical models as mentioned before there is no interpretation of the coefficients to carry out the discussion in this section.**

R: We thank the reviewer for this important comment. In the Discussion section of the revised manuscript, we have clarified the criteria for acceptable or optimal results, providing comparative context when describing the performance of each strain. Terminologies have been harmonized to avoid contradictions, replacing terms such as “more similar”, “equal”, or “higher” with consistent phrasing. Furthermore, the coefficients of the mathematical models have been discussed in detail: for instance, positive coefficients indicate a stimulatory effect of a substrate on biomass production, while negative coefficients highlight limiting or inhibitory interactions. This allows a clear understanding of the contribution of each component (BC, pineapple peels, sugarcane molasses) to bacterial growth and metabolite production.

See Lines 504-529; 680_Revised manuscript unmarked:

**18. The section is totally positive, but what happens with the non-optimal results, because it can be this result, the variability between replicates, the stability of the environment at room temperature, or useful life, with its results, it could be scaled, the cost-benefit**

R: We Thank the reviewer for this comment. Minor differences between replicates were observed, which is expected in triplicate experiments using natural by-products with slightly heterogeneous compositions. Importantly, the carefully optimized formulation ensures reproducible results across all strains. To ensure the reproducibility of the formulated medium, a mathematical formula was established to determine the relative macronutrient contribution of each by-product. This formulation approach allows adjustments of mixture ratios based on the physicochemical characterization of the substrates, ensuring that each preparation maintains a comparable nutritional composition and growth-promoting potential. To further enhance medium stability for industrial applications, dehydration of the medium by oven-drying or lyophilization is planned, as well as cost estimation of the medium on its powder form. This approach is expected to preserve the physico-chemical and biological properties of the medium, allowing long-term storage and easy reconstitution.
