# Supplementary material for: Development of a low-cost culture medium from industrial and environmental by-products for sustainable cultivation of Lactic Acid Bacteria
Source: PLoS One. 2025 Dec 1;20(12):e0337684. doi: 10.1371/journal.pone.0337684 (PMC12668542; doi:10.1371/journal.pone.0337684)
Supplement: Attachment — Submitted filename: Reviewer comments.docx [file pone.0337684.s012.docx]

**Reviewer comments**

The manuscript entitled "**Development of a low-cost culture medium from industrial and environmental byproducts for sustainable cultivation of Lactic Acid Bacteria**", (Manuscript Number: **PONE-D-25-35478**) has been investigated in detail. The manuscript addresses an important and practical topic: developing a sustainable and cost-effective alternative to commercial LAB media (e.g., MRS/M17) using agro-industrial by-products. However, authors need to address some minor points.

I believe the manuscript is suitable for publication in “PLOS ONE”, once the authors have addressed and carried out the minor corrections below.

1. The introduction is well-written but repeats general LAB information that is already well-known. This can be shortened, and more space can be dedicated to gaps in existing low-cost media studies.
2. Grammatical Refinement: Minor grammatical errors occur, such as article misuse (“a medium” vs. “the medium”), which need correction.
3. Avoid Repetition: The phrase “black soldier fly larvae cake” is repeated very frequently—consider abbreviation (e.g., BSFLC) after first mention.
4. Why does the author only consider growth measurements by plate counts (While CFU/mL) is valid, it does not provide insights into growth kinetics or substrate utilization clearly. But inclusion of growth curves (OD600 vs. time), pH reduction over time, or metabolite production (e.g., lactic acid concentration) for better assessment of medium performance.
5. In the Result Section Table 5 Amino Acids Profile of the Various By-products - While the amino acid profile of black soldier fly larvae cake, pineapple peels, and molasses is scientifically relevant, the paper’s main focus is on medium performance (growth and bacteriocin activity) rather than nutrient profiling. The amino acid data are supportive but not critical for understanding the experimental outcomes.
6. Table 8 – Validation of Mathematical Models for Biomass Production - This table contains R², AMDA, and bias factor (Bf) values for the regression models. While important for statistical validation, these are technical details that could be summarized in the main text (e.g., “All models had R² > 0.83 and Bf within 0.75–1.25, indicating well fit”). The full table could go to the supplementary section to declutter the results.

**Final decision to the editor.**

Accept with minor corrections.
